# Metabolic Response of Adult Male Offspring Rats to Prenatal Caffeine Exposure

**DOI:** 10.7759/cureus.7006

**Published:** 2020-02-16

**Authors:** Ioanna Mastroleon, Laskarina-Maria Korou, Vasilios Pergialiotis, Ioannis S Vlachos, Helen Sarlanis, Panagiotis Konstantopoulos, Emmanouil Pikoulis, Despina N Perrea, Nikolaos Kavantzas

**Affiliations:** 1 Laboratory of Experimental Surgery and Surgical Research “N.S. Christeas” (LESSR), National & Kapodistrian University of Athens, Athens, GRC; 2 First Department of Pathology, National & Kapodistrian University of Athens, Athens, GRC; 3 Third Department of Surgery, National & Kapodistrian University of Athens, Athens, GRC; 4 Pathology, National and Kapodistrian University of Athens, Athens, GRC

**Keywords:** caffeine, gestation, rat, lipids, blood pressure

## Abstract

Caffeine is the most widely consumed psychoactive substance, with recommendations from health associations and regulatory bodies for limiting caffeine consumption during pregnancy being increasingly common. Prenatal exposure to caffeine has been shown to increase the risk of developing abnormalities in lipid metabolism in adult life. We further investigated the effect of prenatal caffeine exposure (PCE) (20 mg/kg of body weight) on the metabolic “reserve” of male Sprague Dawley offspring fed on a high fructose diet in adult life. Male adult PCE offspring were assigned to four groups; Nw and Nf: offspring of control mothers (N group of mothers), having received tap water or high fructose water respectively; Cw and Cf: offspring exposed to caffeine during gestation (C group of mothers) and receiving tap water or a high fructose water solution, respectively.

Cf rats presented increased serum triglyceride level, as well as raised systolic and diastolic blood pressure levels, together with extensive renal tissue oedema in adulthood, compared to the other groups (p<0.05 for all comparisons). These findings show further evidence for potential detrimental metabolic effects of prenatal caffeine exposure during adulthood in this animal model.

## Introduction

Caffeine is one of the most widely consumed psychoactive substances throughout the world. The daily consumption of caffeine in the US is estimated to be 211 mg per person [1]. Caffeine readily transcends the placenta barrier and is detected in fetal circulation [2]. During pregnancy, the half-life of caffeine is, on average, 8.3h longer and maybe as much as 16h longer than usual, while in newborns is estimated to be between 65h and 130h [3]. However, pregnant women are usually permitted to consume a moderate dose of caffeine (< 200 mg/d), as it does not appear to increase the risk of miscarriage, congenital malformations, or affect the fetal growth rate [4].

Caffeine has been reported to have anti-obesity and positive metabolic effects when consumed in adulthood [5]. Animal studies have shown that oral administration of caffeine (5 mg/kg of body weight) can increase lipolysis via catecholamine release, and rats fed on a diet with 0.05% caffeine for three or four weeks showed reduced body fat percentage [6,7]. Smaller doses of caffeine (0.005% of food or 0.5 g/kg of food) for eight weeks decreased body fat and systolic blood pressure and improved glucose tolerance and insulin sensitivity after a high-carbohydrate and high-fat diet [8]. Moreover, caffeine intake (1 g/L) for 15 days restored insulin sensitivity and reversed hyperglycemia and hypertension in rats, which consumed a high sucrose diet [9].

On the other hand, the interaction between antenatal caffeine exposure and fetal metabolism remains relatively unknown. A prospective cohort study linked maternal caffeine intake to an 87% increased risk for obesity in the offspring’s adult life [10]. Adult rats prenatally exposed to caffeine (120 mg/kg of body weight) from gestational day 11, which underwent a high-fat diet in adulthood, displayed higher serum glucose levels compared to non-exposed animals [11]. Additionally, in another study, serum insulin levels were lower in adult offspring of female rats exposed to caffeine during gestation and lactation (1mg/mL), compared to control animals [12]. A recent study showed that prenatal exposure to 120 mg/kg of caffeine from gestational day 11 to 20, did not alter the basal blood glucose and insulin, but reduced β cell fraction and mass while increasing increased insulin sensitivity in adult offspring [13]. Finally, prenatal caffeine exposure (PCE) in rats (120 mg/kg) from gestational day 11 until delivery increased the serum levels of total cholesterol and low-density lipoprotein-cholesterol (LDL-C) and decreased the high-density lipoprotein-cholesterol (HDL-C) levels [14]. 

Currently, there are multiple indications that antenatal caffeine exposure has a differential impact on the metabolic profile of fetuses, compared to that of adults. The majority of the studies focused on the baseline levels of the neonates, with a few studies following the offspring to adulthood. Unfortunately, only a handful of studies examine the metabolic tolerance of the offspring against non-ideal diets, which uncover the ability of the organism to sustain metabolic homeostasis, as well as the presence of increased risk for metabolic disorders. A high fructose diet has been shown as a well-controlled and repeatable model of diet-induced metabolic syndrome, with a stronger and more complex phenotype compared to sucrose or starch. Dietary fructose has been shown to induce increased body fat and weight, hyperlipidemia, hypertension, glucose intolerance, and decreased insulin sensitivity, with fructose combining fat and carbohydrate deregulation in both humans and animals. The purpose of the present study is to investigate for the first time the effect of prenatal caffeine exposure on the metabolic profile of adult rats placed under a high fructose diet.

## Materials and methods

Experimental design

Five-month-old female Sprague Dawley rats were matched with males of a respective age (1 male: 2 females). Males were housed with the females for four days to ensure completion of one estrous cycle and were consequently removed. Initiating on entry of the males and up to the day of birth, pregnant rats were administered ad libitum tap water (group N of mothers, control, n=10) or caffeine-enriched water (group C of mothers, n=10), in a concentration estimated to equal a daily caffeine uptake of 20 mg/kg of body weight. The selected dose was comparable to that of the human consumption of approximately two cups of coffee [15,16].

Upon completion of the lactation period (postnatal day 23), the pups were separated from their mothers, and the sex of each offspring was determined. Only male offspring were included in the study since they have been repeatedly shown as prone to metabolic deregulation under high fructose diets [17, 18]. Two or three male rats were randomly selected from each litter. If three males were selected, only two siblings were placed in the same cage and, therefore, in the same group. Upon adulthood (10 weeks old), the selected offspring were assigned to four different subgroups: Nw (n=10), males born of control mothers; Nf (n=10), males born of control mothers, receiving a high-fructose water solution (200 g/l) on a daily basis instead of tap water; Cw (n=10), males prenatally exposed to caffeine; Cf (n=10), males prenatally exposed to caffeine which received a high-fructose water solution (200 g/l) daily instead of tap water.

The administration of a 20% w/v fructose solution to rats starting from the day they turned 10 weeks old (adulthood) for eight weeks was based on the knowledge that this concentration can lead to metabolic changes similar to those of the metabolic syndrome, such as obesity (increased abdominal fat), dyslipidaemia (hypertriglyceridemia), hypertension and hyperglycaemia [19].

**Figure 1:**
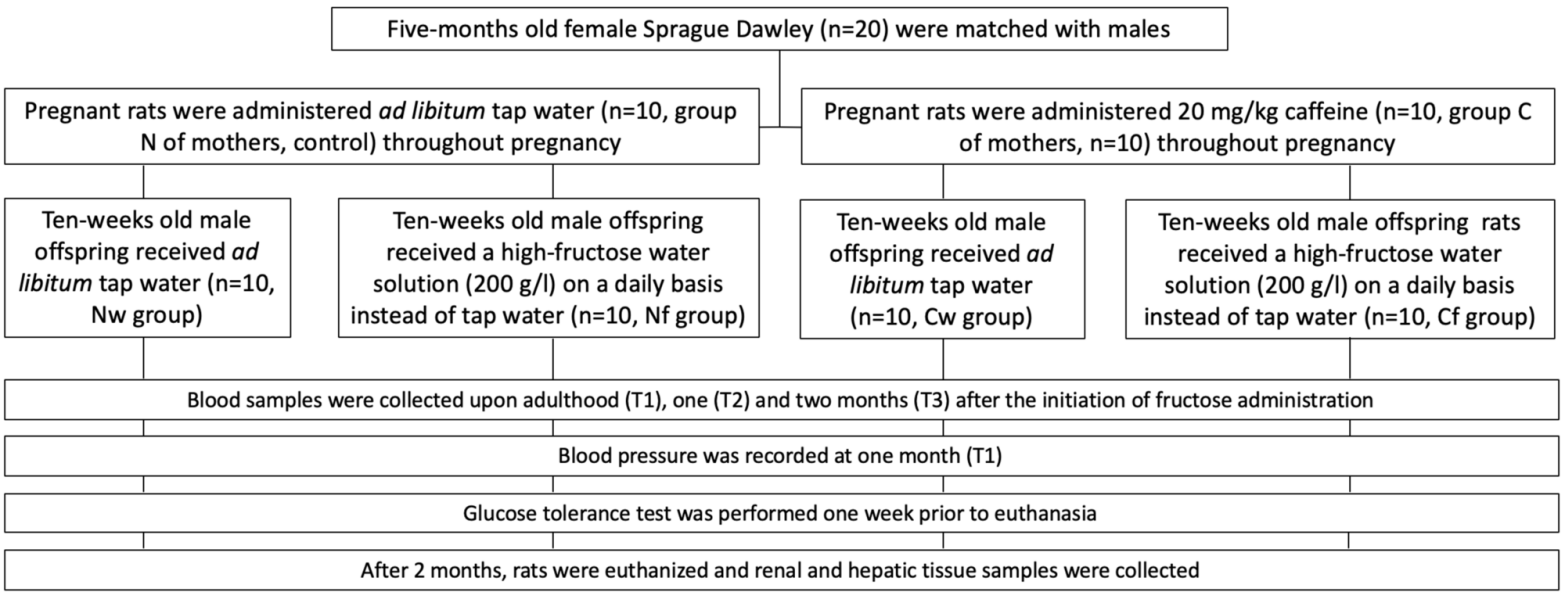
Schematic presentation of the experimental design

The experimental period lasted for two months. At the conclusion of the experiment, the rats were euthanized. Schematic presentation of the experimental design is presented in Figure 1. 

Mothers and offspring were housed under conditions of controlled temperature (23 ± 2^o^C) and humidity (60%), with 12h light/dark cycles. All possible measures were taken to avoid animal suffering at each stage of the experiment. The experimental protocol was reviewed by the Bioethics Committee for studies using animals for scientific purposes and approved by the Veterinary Directorate of Attica Region, in accordance with the EU Directive 2010/63/EE.

The animals had free access to food and water throughout the study. During the study, food and water consumption were recorded daily, while body weight was measured weekly.

Blood sample analyses

Blood samples of male offspring were collected upon adulthood (T1), one month later (T2), and two months (T3). All sample collections were performed at 9:00 am, following a 12-hour fasting period, using capillary tubes introduced into the medial retro-orbital venous plexus under light ether anaesthesia. Serum was separated by centrifugation at 3000 rpm for 10 min and was stored at -80°C awaiting analysis.

Serum concentrations of total cholesterol and triglycerides were determined using an enzymatic phenol-aminophenazone (PAP) commercial kit ("Biosis" - Biotechnological Applications, Athens, Greece), whereas HDL-cholesterol was determined with a cholesterol enzymatic photometric method. LDL-cholesterol was determined using the Friedewald formula. Serum glucose levels were measured by colorimetric method.

Blood pressure measurements

Systolic and diastolic blood pressure was recorded by using a non-invasive tail-cuff blood pressure system (Coda 2, Kent Scientific, U.S.A.) at one month, following the high fructose feeding between 10 am and 2 pm. The animals were familiarized with the blood pressure measuring equipment before initiation of the measurements. For each rat, three blood pressure measurements were recorded, and their average was registered as their blood pressure measurement.

Glucose tolerance test (GTT)

The glucose tolerance test was performed one week before euthanasia. After a 16h, overnight fasting period, blood samples were collected from the dorsal pedal vein of the rats, and fasting serum glucose levels were determined by glucometer. The animals were weighed to estimate glucose doses (1.5 g of glucose/kg body weight). The rats were then injected (intraperitoneal) with the glucose solution, and subsequently, blood was collected from the dorsal pedal vein of the animals at 30 min, 60 min, and 120 min to measure serum glucose levels.

Histopathology analysis of renal and hepatic tissue samples

The specimens were fixed in 10% formaldehyde for 24 hours. Paraffin-embedded sections of 4 μm depth were stained with hematoxylin and eosin. All sections were examined by light microscopy at different magnifications (x50, x100).

Statistical analysis

Quantitative variables are described as a median [interquartile range]. The Kruskal-Wallis test was implemented for multiple group comparisons, whereas the Mann-Whitney U test was used for post hoc multiple testing. In all cases of multiple hypothesis testing, Benjamini-Hochberg's false discovery rate (FDR) was applied to assess differences and control family-wise errors as <0.05. All tests were two-sided. P<0.05 was indicative of a statistically significant difference.

## Results

No differences in litter size and sex ratio of the offspring were observed. Each litter comprised an average of 10 pups. The number of offspring was 100 in total (51 female and 49 male) for group N and 96 (46 female and 50 male) for group C, without a significant difference. There was a significant difference, however, in the birth weight of offspring between groups N and C, with caffeine-administered mothers having lower-weight offspring (p<0.001) (Group N: 55 g (50-64 g), Group C: 46 g (44-48 g)).

Regarding the glucose tolerance test, Nf and Cf animals had higher median glucose levels at 30’, 60’, and 120’ of the glucose tolerance test, but the differences were not identified as statistically significant. Also, there was no significant difference in fasting glucose levels among groups at any of the time points, but there was a significant difference for the Nw, Nf, Cw, and Cf groups in the different time points (Table 1).

**Table 1:** Serum glucose levels (mg/dl) recorded following the glucose tolerance tests (GTT0, GTT30, GTT60, GTT120) and at T1, T2 and T3; and serum levels of total cholesterol (mg/dl), HDL cholesterol (mg/dl), LDL cholesterol (mg/dl), triglycerides (mg/dl) and blood pressure (systolic, diastolic and mean blood pressure) levels per animal group studied (Nw, Nf, Cw, Cf) at T1, T2, and T3 Data are expressed as median (range). P-values marked with (*) show a statistically significant difference (p<0.05). Post hoc analysis to determine differences among groups for the Kruskal-Wallis analysis: Figures sharing the same superscript letters differentiate significantly from each other. TGL (T2): 1) Nw vs Nf P=0.025, 2) Nw vs Cf p=0.001, 3) Nf vs Cw p=0.045, 4) Cw vs Cf p=0.004 TGL (T3): 1) Nw vs Nf p=0.015, 2) Nw vs Cf p=0.001, 3) Nf vs Cw p=0.002, 4) Nf vs Cf p=0.037, 5) Cw vs Cf p<0.001 LDL (T2): 1) Nw vs Cw p=0.052, 2) Nw vs Cf p=0.015, 3) Cw vs Cf p=0.001 Systolic blood pressure: 1) Nw vs Nf p<0.001, 2) Nw vs Cw p<0.001, 3) Nw vs Cf p<0.001, 4) Nf vs  Cw p=0.025, 5) Nf vs Cf p=0.019, 6) Cw vs Cf p<0.001 Diastolic blood pressure: 1) Nw vs Nf p=0.002, 2) Nw vs Cf p<0.001, 3) Nf vs Cw p=0.041, 4) Nf vs Cf p=0.053, 5) Cw vs Cf p=0.005 Weight (T3): 1) Nf vs Cw p=0.041, 2) Cw vs Cf p=0.005 Nw group - offspring of control mothers receiving tap water Nf group - offspring of control mothers receiving high fructose water Cw - offspring exposed to caffeine during gestation receiving tap water Cf - offspring exposed to caffeine during gestation receiving high fructose water GTT -  glucose tolerance test; HDL - high-density lipoprotein; LDL - low-density lipoprotein

Variable	Nw group	Nf group	Cw group	Cf group	p-value (Kruskal-Wallis)
GTT 0	100 (85-122)	95 (71-129)	105.5 (58-131)	108 (61-130)	0.829
GTT 30	171.5 (122-281)	223 (176-321)	215 (95-386)	210.5 (134-352)	0.349
GTT 60	129 (90-162)	151.5 (114-201)	124 (95-283)	175.5 (100-220)	0.089
GTT 120	111 (83-123)	120 (84-147)	99 (84-189)	114.5 (84-145)	0.337
Glucose T1	140 (130-165)	145 (120-200)	147.5 (120-170)	147.5 (120-170)	0.731
Glucose T2	150 (140-180)	155 (120-175)	155 (125-180)	157.5 (145-190)	0.607
Glucose T3	177.5 (135-220)	180 (138-230)	187.5 (165-210)	195 (168-240)	0.401
p-value (Friedman)	0.001	0.007	<0.001	0.001	
Total cholesterol (T 1)	99.5 (85-110)	105 (90-120)	95 (80-110)	97.5 (80-115)	0.171
Total cholesterol (T2)	82.5 (70-100)	82.5 (70-120)	82.5 (80-100)	80 (65-90)	0.473
Total cholesterol (T3)	75 (60-100)	85 (65-100)	80 (65-95)	75 (65-95)	0.705
p-value (Friedman)	0.001	0.013	0.002	<0.001	
Triglycerides (T1)	63 (45-95)	70 (50-126)	77.5 (60-120)	80 (60-140)	0.254
Triglycerides (T2)	87.5 (65-115) ^a,b^	112.5 (60-195) ^a,c^	82.5 (45-150) ^c,d^	140 (90-190) ^b,d^	0.002 *
Triglycerides (T3)	70 (40-105) ^a,b^	97.5 (60-150)^ a,c,d^	65 (40-80) ^c,e^	150 (80-240) ^b,d,e^	<0.001 *
p-value (Friedman)	0.053	0.266	0.020	0.012	
HDL (T1)	53 (46-61)	56 (48-65)	51.5 (45-59)	53 (46-60)	0.284
HDL (T2)	54 (46-60)	55.5 (49-66)	75 (46-150)	56.5 (47-190)	0.572
HDL (T3)	54 (46-60)	54.5 (48-65)	50.5 (40-58)	53 (46-59)	0.249
p-value (Friedman)	0.168	0.053	0.001	0.010	
LDL (T1)	31 (21-37)	35 (14-43)	29 (11-38)	60 (6-36)	0.269
LDL (T2)	10 (5-22) ^a,b^	10 (1-38)	17 (8-31) ^a,c^	5 (2-17) ^b,c^	0.013 *
LDL (T3)	10 (0-21)	5 (2-26)	16 (4-22)	12.5 (0-24)	0.328
p-value (Friedman)	0.001	0.007	0.050	0.023	
Systolic blood pressure	135 (122-143)^ a,b,c^	152 (148-162) ^a,d,e^	144 (140-155) ^b,d,f^	160.5 (152-174) ^c,e,f^	<0.001
Diastolic blood pressure	106 (80-117) ^a,b^	120.5 (107-139) ^a,c,d^	106 (88-138) ^c,e^	130 (112-143) ^b,d,e^	<0.001
Weight (T1)	336 (296-360)	337 (296-378)	317 (264-352)	344 (310-380)	0.131
Weight (T2)	414 (378-450)	400 (366-458)	388 (348-410)	403 (392-456)	0.148
Weight (T3)	444 (404-460)	446 (370-512) ^a^	418 (368-456) ^a,b^	442 (406-494) ^b^	0.042 *

No significant differences were observed between the groups at T1 measurements regarding serum lipid levels (Table 1). At T2, animals in group Nw had lower serum triglyceride levels in comparison with animals of both Nf (P=0.025) and Cf (P=0.001) groups. Triglyceride levels in group Cw were lower than the respective levels in Nf (P=0.045) and Cf (P=0.004) groups. Serum LDL cholesterol levels analysis at T2 revealed that animals in Cw group had increased levels in comparison with LDL cholesterol levels in group Nw (P=0.052) and group Cf (P=0.001), with animals in group Nw displaying higher such levels than those in group Cf (P=0.015). At T3, animals in group Nw had lower triglyceride levels than animals in groups Nf (P=0.015) and Cf (P=0.001). Animals in group Nf had higher such levels than those in group Cw (P=0.002), but lower triglyceride levels than Cf animals (P=0.037). Moreover, group Cw animals had lower triglyceride levels than animals of group Cf (P<0.001).

Comparison of systolic blood pressure measurements between the groups showed that animals of groups Nw and Cw had a lower systolic pressure when compared to those recorded in animals of groups Nf (Nw vs Nf and Cw vs Nf, P<0.001 and P=0.025 respectively) and Cf (Nw vs Cf, P<001; Cw vs Cf, P<0.001). Also, rats in Nw and Nf groups had lower systolic blood pressure than animals in Cw and Cf groups respectively (Nw vs Cw, P<0,001; Nf vs Cf, P=0.019). Diastolic blood pressure was higher in group Nf in comparison to group Nw (P=0.002), and in group Cf compared to Cw (P=0.005). Statistical analysis also indicated increased diastolic blood pressure measurements in group Cf compared to group Nf (P=0.053), and lower values in group Nw when compared to group Cf (P<0.001). Finally, group Nf had higher diastolic pressure than group Cw (P=0.041) (Table 1).

Significant differences in body weight were recorded at the end of the experimental period between groups. Specifically, Cw group had lower body weight levels compared to Nf (P=0.041) and Cf (P=0.005) groups. However, there were no differences identified between the two control offspring group (Nw, Cw). Fructose-diet groups had significantly less food consumption vs control diet groups, since they received extra calories from the fructose added in the water. At T2 and T3, Nf and Cf animals increased water consumption compared to T1, leading to receiving higher amounts of fructose during the final month of the experiment. Importantly, no differences in food and water consumption were identified between the two control diet groups (Nw vs Cw) or between the two fructose diet groups (Nf vs Cf) (Table 2).

**Table 2:** Food consumption (g/day) and water intake (ml/day) per animal group studied at T1, T2, and T3 Data are expressed as median (interquartile range). Post hoc analysis to determine differences among groups for the Kruskal-Wallis analysis: Figures sharing the same superscript letters differentiate significantly from each other. Food Consumption T1: Nw vs Cf, P=0.002; Cw vs Cf, P<0.001 T2: Nw vs Nf, P=0.03; Nw vs Cf,  P<0.001; Cw vs Cf,  P<0.001 T3: Nw vs Nf, P<0.001; Nw vs Cw, P=0.012; Nw vs Cf,  P<0.001; Cw vs Cf, P<0.001 Water intake T1: Nw vs Nf, P<0.001; Cw vs Cf, P<0.001; Nf vs Cw, P<0.001 T2: Nw vs Nf, P<0.001; Cw vs Cf, P<0.001; Nf vs Cw, P<0.001; Nw vs Cf, P<0.001 T3: Nw vs Nf, P<0.001; Cw vs Cf, P<0.001; Nf vs Cw, P<0.001; Nw vs Cf,  P<0.001 Nw group - offspring of control mothers receiving tap water Nf group - offspring of control mothers receiving high fructose water Cw - offspring exposed to caffeine during gestation receiving tap water Cf - offspring exposed to caffeine during gestation receiving high fructose water

		Nw	Nf	Cw	Cf
Food Consumption (g/day)	T1	31 (11) ^a^	31 (18.2)	28 (5) ^b^	15 (3.2) ^a,b^
	T2	27 (2) ^a,b^	26 (10.5) ^a^	26 (2.25) ^c^	16 (6) ^b, c^
	T3	27 (2.75) ^a,b^	14 (5.25) ^a^	25 (2) ^c^	14.5 (1.5) ^b,c^
Water Intake (ml/day)	T1	34.5 (5.75) ^a^	52 (25.25) ^a,b^	34.5 (1.5) ^b,c^	57 (8.25) ^c^
	T2	34 (4.75) ^a,b^	85 (21.75) ^a,c^	33 (6.75) ^c,d^	84 (6.5) ^b,d^
	T3	34 (1.75) ^a,b^	84 (18) ^a,c^	35.5 (6.25) ^c,d^	84 (18.5) ^b,d^

**Figure 2:**
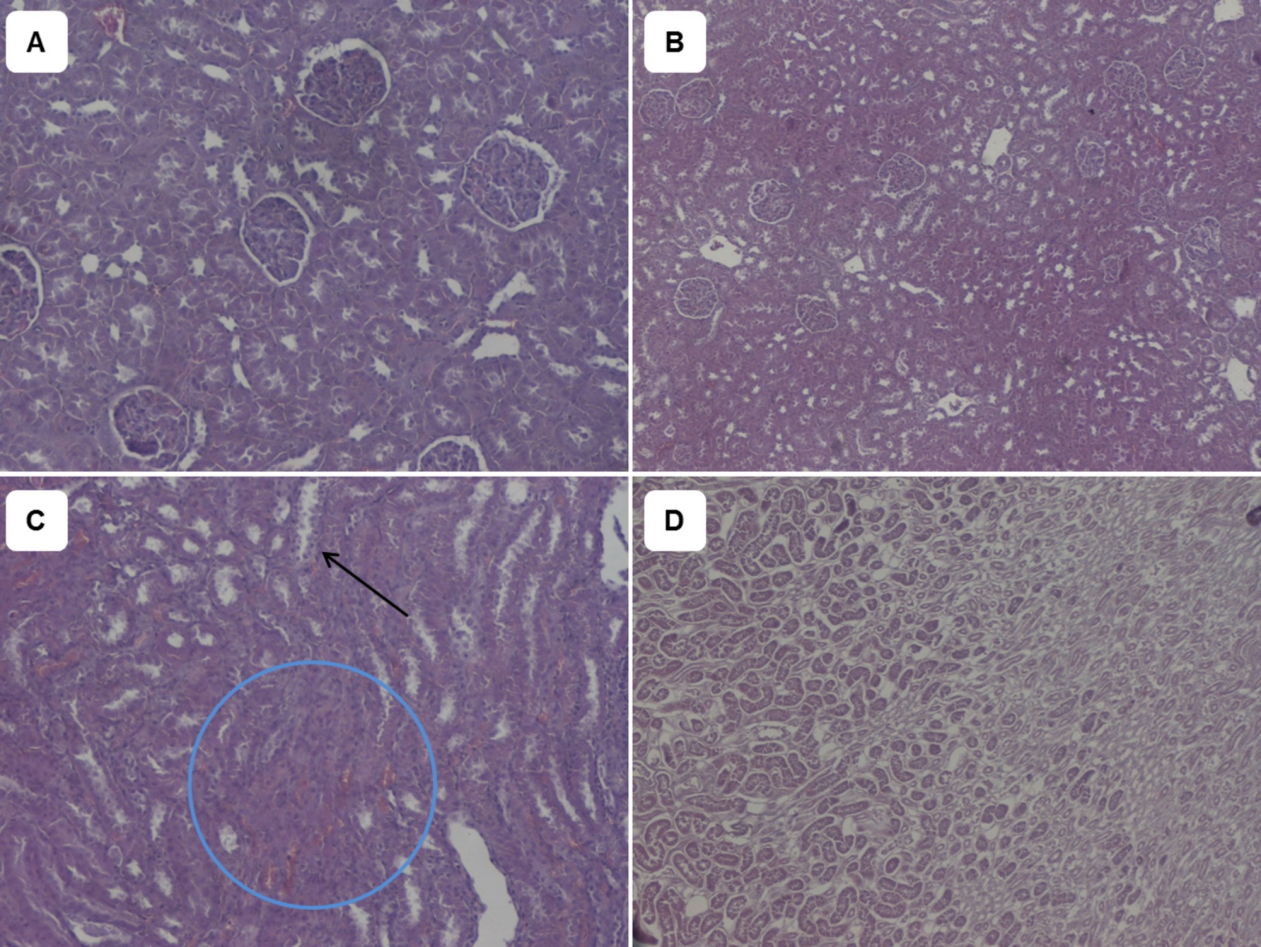
Haematoxylin/Eosin stains of renal tissues A. Males born of control mothers which received tap water; B. Males born of control mothers, which received a high fructose water solution (200 g/l) daily instead of tap water; C. Males prenatally exposed to caffeine which received tap water; D. Males prenatally exposed to caffeine which received a high fructose water solution (200 g/l) daily instead of tap water. The arrow in Figure C indicates hydropic degeneration of tubular epithelium. The encircled area in Figure C and the whole area in Figure D present tissue with interstitial oedema.  (A, C: 100x, B, D: 50x)

No differences were observed in renal or hepatic tissue swelling between groups. However, hematoxylin-eosin stained renal tissue samples obtained from Cw and Cf groups revealed a statistically significant higher number of cases (50% in group Cw and 70% in group Cf) with oedema, compared to group Nw (Nw vs Cw, P=0.033 and Nw vs Cf, P=0.003) (Table 3, Figure 2).

**Table 3:** Analysis of histopathologic examination of renal and hepatic tissue samples, in relation to the presence or absence of oedema or swelling Figures sharing the same superscript letters differentiate significantly from each other. Renal tissue oedema: Nw vs Cw, P=0.033  and Nw vs Cf, P=0.003 Nw group - offspring of control mothers receiving tap water Nf group - offspring of control mothers receiving high fructose water Cw - offspring exposed to caffeine during gestation receiving tap water Cf - offspring exposed to caffeine during gestation receiving high fructose water

Lesion in renal or hepatic tissue		Number of animals per group presented as percentage (%)			
		Nw (%)	Nf (%)	Cw (%)	Cf (%)
Oedema in renal tissue	Presence	0^a,b^	40	50^a^	70^b^
Swelling in renal tissue	Presence	30	10	50	30
Oedema in hepatic tissue	Presence	0	0	10	10
Swelling in hepatic tissue	Presence	0	0	0	0

## Discussion

In our study, we observed elevated triglyceride levels in male rats prenatally exposed to caffeine and having received a high fructose diet in adulthood (group Cf) compared to all other the experimental groups, including group Nf (offspring of mothers not receiving caffeine but under high fructose diet). This observation directly implicates prenatal caffeine exposure in metabolic homeostasis during adulthood. Similar findings were identified in previous studies where rats prenatally were exposed to caffeine (120 mg/Kg of body weight) and received a high-fat diet in adulthood [11, 20]. Both studies support the hypothesis that such a diet triggers excessive lipogenesis in rats prenatally exposed to caffeine, the latter appearing to be particularly susceptible to develop metabolic syndrome. One of the studies also supported that this effect eventually leads to hepatic macrovesicular steatosis [20]. This finding was not established in our study, perhaps due to the lower dose of caffeine administered during the gestational period (20 mg/kg/day). Nevertheless, all studies, including ours, show clear metabolic deterioration following antenatal caffeine exposure during adulthood using two different metabolic syndrome models (high-fat diet and high fructose diet).

Food consumption was significantly lower in fructose groups Nf and Cf, compared to control diet groups Nw and Cw. This may be attributed to higher fluid consumption in the fructose groups, which, as previously observed, leads to an additional calorie intake via the fructose solution [19]. Furthermore, the sweet-tasting fructose enhanced the palatability of the solution and was thus preferred by the rats over the standard chow. Importantly, Nw and Cw (control diet groups), as well as Nf and Cf (fructose diet groups), had similar food and water consumption levels (Nw vs Cw, and Nf vs Cf).

Studies in adult rats have shown favorable outcomes of caffeine administration in connection with insulin sensitivity, blood glucose, and hypertension [9]. In the study of Conte et al., 1g/L of caffeine was administered to adult rats via drinking water for 15 days. Οn the contrary, in our study, the administration of caffeine occurred during intrauterine life, possibly affecting organ development, as well as creating a different environment inducing epigenetic changes and offering different possible explanations for the differences observed.

In this study, prenatally caffeine exposure (PCE) in rats’ offspring led also to raised systolic blood pressure compared to the control group. The difference was even more profound when combined with a high fructose diet. To our knowledge, this is the first study to indicate that maternal caffeine consumption may increase the susceptibility of male offspring to hypertension. However, the underlying mechanism remains so far undetermined, and therefore, further research is required.

A potential pathophysiologic pathway, possibly triggering this effect, appears to be that of renal developmental abnormalities caused by PCE, including glomerulosclerosis and interstitial sclerosis [21]. Glomerulosclerosis is a glomerular lesion that leads to a progressive deterioration in renal function. In the aforementioned study, histological examination of tissue samples harvested from adult offspring antenatally exposed to caffeine revealed pathological features and alterations in renal architecture. Structural damage was identified in podocytes, a group of highly differentiated cells responsible for the maintenance of the glomerular basement membrane, accompanied by a reduction in podocyte markers, most importantly that of renal angiotensin II receptor type 2 (AT2R) gene expression. PCE offspring kidneys exhibited an enlarged Bowman’s space and significantly reduced glomerular tuft and cortex width, as well as an increased nephrogenic to cortical zone ratio. The authors proposed that the compromised AT2R functional programming observed in the PCE group may partially mediate the developmental glomerulosclerosis in adults, which in turn generates hypertension. Furthermore, the renin-angiotensin system (RAS) is crucial to kidney development. Therefore, any potential impairment in this system could lead to renal morphological abnormalities or dysfunction. A study in rats showed that a dose of 180 mg/kg per day of caffeine during pregnancy increased maternal plasma Angiotensin-II levels, leading to chronic activation of the maternal and placental RAS [22]. Other animal experiments suggested that prenatally developed renal dysplasia leads to high susceptibility to renal diseases [23-25]. In our study, both caffeine exposure groups (Cw and Cf) had higher frequencies of adult renal oedema (50% in group Cw, and 70% in group Cf) compared to the control groups.

We also observed higher systolic blood pressure in the caffeine exposure groups compared to Nw and Nf groups. Furthermore, we also observed hypertriglyceridemia in both caffeine groups compared to controls, showing multiple constituents of the metabolic syndrome being affected by antenatal caffeine exposure. It has been shown previously that caffeine consumption during pregnancy resulted in elevated levels of maternal glucocorticoids, which inhibit the activity of the fetal hypothalamic-pituitary-adrenal (HPA) axis via a negative feedback regulation [26]. This impairment led to intrauterine growth restriction (IUGR) and increased the risk of developing metabolic syndrome in adulthood. The findings in our study indicate a similar susceptibility in developing metabolic syndrome, as shown by the high levels of triglycerides and raised blood pressure. 

Literature about human fetuses exposed to caffeine showed that children whose mothers consumed 4-5.9 and ≥6 units of caffeine per day (1 unit=90 mg caffeine) during pregnancy tended to have a higher childhood body mass index and total body fat mass [27].

## Conclusions

The results of our study clearly show phenotypic effects of PCE in the development of metabolic syndrome in adulthood. Certainly, as in all experimental studies, the results have to be viewed under a translational lens and not directly transferred to humans. Nevertheless, our study is the first to examine the effects of PCE in a model of adult metabolic syndrome induced by a high fructose diet. We observed significant interaction of PCE with the presence of lower birth weight, high blood pressure, renal oedema, and hypertriglyceridemia. Taking into account that caffeine doses used in our study were well within the accepted range for human pregnancies, as well as how widespread coffee consumption is during pregnancy, these findings at least provide significant motivation for further studies.
